# Going out for dinner—The consumption of agriculture pests by bats in urban areas

**DOI:** 10.1371/journal.pone.0258066

**Published:** 2021-10-21

**Authors:** Ludmilla M. S. Aguiar, Igor D. Bueno-Rocha, Guilherme Oliveira, Eder S. Pires, Santelmo Vasconcelos, Gisele L. Nunes, Marina R. Frizzas, Pedro H. B. Togni

**Affiliations:** 1 Laboratory of Bat Biology and Conservation, Department of Zoology, University of Brasília, Brasília, Distrito Federal, Brazil; 2 Ecology Graduate Course, Department of Zoology, University of Brasília, Brasília, Distrito Federal, Brazil; 3 Instituto Tecnológico Vale—Desenvolvimento Sustentável, Belém, Pará, Brazil; 4 Laboratory of Biology and Ecology of Coleoptera, Department of Zoology, University of Brasília, Brasília, Distrito Federal, Brazil; 5 Laboratory of Insect Ecology, Department of Ecology, University of Brasília, Brasília, Distrito Federal, Brazil; Instituto Federal de Educacao Ciencia e Tecnologia Goiano - Campus Urutai, BRAZIL

## Abstract

Insectivorous bats provide ecosystem services in agricultural and urban landscapes by consuming arthropods that are considered pests. Bat species inhabiting cities are expected to consume insects associated with urban areas, such as mosquitoes, flying termites, moths, and beetles. We captured insectivorous bats in the Federal District of Brazil and used fecal DNA metabarcoding to investigate the arthropod consumed by five bat species living in colonies in city buildings, and ascertained whether their predation was related to ecosystem services. These insectivorous bat species were found to consume 83 morphospecies of arthropods and among these 41 were identified to species, most of which were agricultural pests. We propose that bats may roost in the city areas and forage in the nearby agricultural fields using their ability to fly over long distances. We also calculated the value of the pest suppression ecosystem service by the bats. By a conservative estimation, bats save US$ 94 per hectare of cornfields, accounting for an annual savings of US$ 390.6 million per harvest in Brazil. Our study confirms that, regardless of their roosting location, bats are essential for providing ecosystem services in the cities, with extensive impacts on crops and elsewhere, in addition to significant savings in the use of pesticides.

## Introduction

Although bats make up almost half of the mammalian fauna in some localities in Latin American countries, they are frowned upon because of their association with diseases, including rabies transmission [1]. The negative image of bats is overshadowing their critical roles in agriculture. They disperse seeds, pollinate plants, and perform ecosystem services such as suppressing biting insects and agricultural pests [2], contributing directly to the economy [3].

However, approximately 15% of the bat species are threatened [4] due to land-use change, hunting and persecution, quarrying, habitat intrusions, and urbanization. In addition, the application of pesticides causes irreversible genetic damages and long-term sublethal effects on the insectivorous bat populations [5, 6]. Moreover, the negative perception of bats increases the persecution and extermination of bats worldwide, hindering the efforts to conserve their declining populations.

Brazil, for example, harbors a rich bat fauna, but its economy relies heavily on the agribusiness. Like in several other countries, Brazilian bat fauna also experiences negative pressures. Contributing to approximately 30 and 15% of the global soybean and meat production, respectively, Brazil is also a leading exporter of sugar, chicken, and coffee. Agricultural production accounts for more than 20% of Brazil’s gross domestic product (GDP) [7]. At the same time, Brazil is the world’s largest consumer of pesticides. In 2018, Brazil used 377,176 tons of pesticides, of which an amount worth US$ 3 billion was imported [7]. It is important to call attention that foraging over crops may lead to increased bats exposure to pesticides [8].

More than half (52%) of Brazil’s soybean is produced in the Cerrado biome [9]. The 15.6 million hectares (Mha) of soybean represent 90% of all the agricultural crops grown in the Cerrado biome. Also, planted pasturelands account for 76 Mha of the Cerrado. On the other hand, the Cerrado domain in central Brazil is a global biodiversity hotspot [10]. It harbors more than 4,800 plant species and 1,600 species of mammals, birds, and reptiles. There are at least 118 bat species in the Cerrado, accounting for 66.3% of all the bat species recorded in Brazil, 10.5% of all the bat species recorded globally, and 47.0% of all the mammals inhabiting the Cerrado [11]. Like other developing countries, Brazil is also undergoing rapid urbanization. Urban growth mainly occurs in the territories adjacent to cities (peri-urban areas) where agricultural activities are still present [12]. Although in literature Brazil may be considered successful in achieving sustainable urbanization [13] its largest urban centers are located at the critical regions of biodiversity in the Atlantic Forest and Cerrado biomes, and the amount of green urbans areas is frequently below the world’s average. Moreover, the urban fauna represents only a small fraction of the native fauna present in natural areas.

Bats are the most abundant mammals present in urban centers [14], but even so there are constant requests from the residents for their removal from the voids, ceilings, and other cavities in buildings. In the cities, bats can fly in open spaces, have plenty of roosts, and likely benefit from a large number of insects attracted by artificial light [14]. Insectivorous bats may play an important role as biological control agents in agricultural lands undergoing urbanization, regulating the populations of agricultural pests in rural environments and disease vectors in urban environments and providing essential ecosystem services in the urban landscape.

However, the data on the biology and ecology of the insectivorous bats inhabiting these cities are scarce. Most studies on urban bats have been conducted in the Northern Hemisphere. Vegetation is indicated to be important in maintaining insect prey populations [15], while artificial lights can improve the prey capture rate of the bat species adapted to hunting in bright places [16]. Several studies have suggested the suppression of arthropods, including agricultural pests and disease vectors, by urban bats [17, 18]. However, insectivorous species are generally poorly sampled since many studies on neotropical bats have been conducted on species more easily captured by mist nets. Although insectivorous bats occupy natural areas and some species, especially those of the Molossidae, seem to be well-adapted to urban areas, little is known about them.

We believe that identifying relevant bat species and their ecosystem services will help design biodiversity-friendly urban landscapes. The lack of information on the role of insectivorous bats in cities and anywhere will impair the development of strategies for maintaining biodiversity and ecosystem services [19], protecting the coexistence of bats and humans in the same tropical habitat.

With rapid urbanization in Brazil, it will be interesting to assess how insectivorous species live in cities and whether they continue to provide ecosystem services by preying on pests like mosquitoes, such as *Aedes aegypti* (Diptera: Culicidae), a known vector of viruses including dengue, chikungunya, and Zika viruses [20]. Therefore, thorough knowledge of the bats’ diet will help determine the role of the bats in cities, i.e., whether and what type of ecosystem service they provide. In addition, DNA metabarcoding is a tool to explain their importance [21].

This study aimed to identify arthropod species preyed upon by insectivorous bats in cities and investigate whether bat predation was related to the provision of ecosystem services. We first examined how the diet of five urban insectivorous bat species varied and analyzed the main functional groups of insects on which they preyed. We hypothesized that urban insectivorous bats complemented each other by preying on different groups of insects. We also hypothesized that the bats mostly preyed on synanthropic species, such as flies and mosquitoes. We tested these hypotheses by collecting the feces from five bat species captured in the colonies established in the buildings in the Federal District of Brazil. The collected feces were analyzed with DNA metabarcoding to identify the arthropods consumed by the urban insectivorous bats. Furthermore, we estimated the value of the ecosystem service provided by bats through the consumption of a major agricultural pest.

## Materials and methods

### The study area

**Fig 1:**
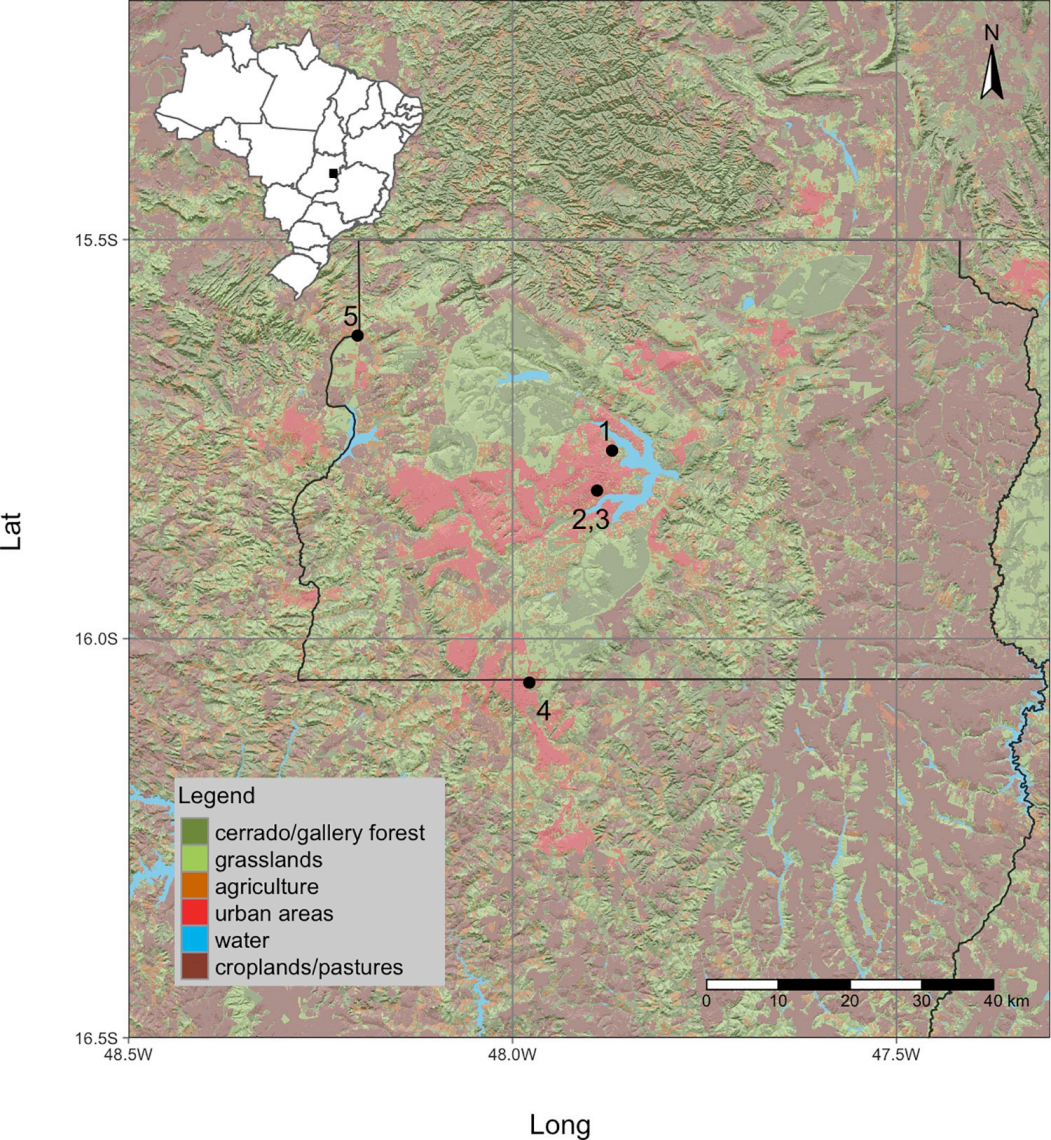
Map showing the location of bat ocolonies and land use in the Federal District of Brazil. 1 = *Nyctinomops laticaudatus; 2 = Cynomops planirostris; 3 = Molossus molossus; 4 = Eumops perotis; 5 = Histiotus diaphanopterus*.

All the urban areas we studied here were within the Cerrado domain (Fig 1). The bats from five colonies found in the urban areas of Brasília, Padre Bernardo, and Valparaíso were sampled. Brasília is Brazil’s federal capital. Valparaíso of Goiás is located on the plateau known as Planalto Central, state of Goiás, southwest of Brasília. Padre Bernardo is a municipality located 42 km north of the boundary of the Federal District in Goiás, Brazil. The colonies included an *Nyctinomops laticaudatus* colony in the University Restaurant of the University of Brasília (15° 45′ 51.6′′ S, 47° 52′ 13.1′′ O), a *Cynomops planirostris* colony and a *Molossus molossus* colony in the commercial block 405 Sul, Brasília (15° 48′ 51.9′′ S, 47° 53′ 23.4′′ O), a *Eumops perotis* colony in a building in Valparaíso-GO (16° 03′ 18.4″ S, 47° 58′ 41.6″ O), and a *Histiotus diaphanopterus* colony in Residential Vendinha, Padre Bernardo-GO (15° 37′ 12.9″ S, 48° 12′ 06.5″ O).

### Bat capture and feces collection

We captured the bats with mist nets opened at the exit of each colony for three nights each in March (rainy season) and July (dry season) of 2018. Nets were kept opened from 6 PM to 6 AM. The bats were captured at the exits of the shelters throughout the night with the same type of mist net and the same sampling effort between the two stations. Since it was impossible to use mist nets on the tops of the buildings, hand nets were used to capture the bats at the *E*. *perotis* colony. Bats were identified using Dias et al. [22]. One specimen of each species was deposited in the Mammal Collection at the University of Brasília under numbers CCUNB0894—*Nyctinomops laticaudatu*s, CCUNB 1403—*Molossus molossus*, CCUNB 1404—*Histiotus diaphanopterus*, CCUNB 1405—*Cynomops planirostris* and CCUNB 1407—*Eumops perotis*. Bats captured when leaving the colony were weighed, banded, and released at the same site. Bats captured returning to the colony, were kept in cotton bags for 30 min to defecate before being re-released. After defecating in a clean cotton bag, pellets were collected with sterile forceps and transferred to 2 ml tubes. They were stored dry using silica [23] overnight, therefore at lower temperatures. Upon arrival at the laboratory at dawn, the tubes were stored at -20°C until the moment of extraction. This study was submitted and approved by the Ethics Commission on Animal Use at the University of Brasília (CEUA/UnB) (process #116319/2011). Captures in Protected Areas were permitted by Chico Mendes Institute for Biodiversity Conservation (ICMBio/MMA) through license number 39296–1.

### Metagenomics

Upon returning to the laboratory, we immediately stored the pellets at -20°C. All the fecal pellets ranging from 0.002–0.330 g of feces per sample were used for DNA extraction. The extraction was carried out in Brasília-DF, at the University of Brasília, and the other steps in the Laboratory of Instituto Tecnológico Vale, in Belém-PA. Both laboratories do not conduct DNA metabarcoding experiments. The pellets were cut and macerated with a scalpel previously sterilized in 96% ethanol and with an open flame. There was an update of the extraction kit by the manufacturer. One kit was purchased before and one after this update. Thus, half of the extractions were performed using the QIAamp DNA Stool Mini Kit (Qiagen, Hilden, Germany) according to the manufacturer’s instruction and with the modifications described in Zeale’s paper [24]. The other half of the extractions were performed using the QIAamp Fast DNA Stool Mini Kit, according to the manufacturer’s protocol. The fecal DNA was amplified using two pairs of generic primers, UEA3 and UEA4 and UEA5 and UEA6 [25] for the mitochondrial cytochrome c oxidase subunit I (COI), producing amplicons of approximately 370 base pairs (bp) and 350 bp [26] respectively. These are primer pairs that have been extensively tested and have worked well at the Instituto Vale’ laboratory. The PCR amplification was carried out in 25-μL reactions, containing 8 μL of sample DNA, 5 μL of 5X MyTaq ™ Reaction Buffer Colorless, 2 μL of MgCl_2_, 2 μL of each primer, 0.125 μL of Taq DNA polymerase, 0.375 μL of ultrapure water, 2 μL of dNTPs, and 5 μL of tributyltin (TBT). The PCR underwent an initial denaturation at 95°C for 5 min, followed by 35 cycles of denaturation at 95°C for 1 min, annealing at 48°C for 1 min, and extension at 72°C for 1 min and 30 s, and a final extension of 7 min at 72°C before storage at 4°C [26].

Two μL of the PCR product were run on an agarose gel at 120 V for 30 min and stained with Sybr Safe® to verify the quality of the amplified bands [26]. The samples with unnoticeable bands were excluded from further analysis. Next, the samples were purified twice with a short PCR step, using eight base tags attached to the ends of the primers to individualize each sample in a multiplexed library model, following the 16S metagenomic sequencing library preparation protocol recommended for preparing samples for sequencing on the MiSeq Illumina Sequencer (Illumina, Inc., San Diego, CA, USA). At all stages, a control sample was generated to visualize the possible effects of contamination on the PCR products that underwent multi-amplification. The quality check of all the amplicons was performed by quantifying their DNA concentration using Qubit® and fragment size using TapeStation®. The PCR products labeled with the tags were then pooled. Negative controls, which were also labeled, were included in the pool when some DNA was detected by Qubit®.

The sequencing of the amplicons was performed using the Illumina MiSeq® platform, an open-system platform using the amplicons tools software package, and run according to the manufacturer’s. The Illumina paired-end command was executed to pair the reading pairs (forward and reverse) in a complete fragment overlapping their 3′-reading ends. Demultiplexing and primer removal was performed using the *ngsfilter* command. The *obiuniq* command de-replicated the readings and determined the number of repetitions of each reading per sample. Of these unique sequences, only those that appeared with a minimum abundance of 10 repetitions were retained [27, 28]. This filter was performed with the *obigrep* function, eliminating the singletons (sequences with one repetition) and possible PCR and sequencing errors. Using the formula R = Ae/Ao, with Ae as the abundance of wrong sequences and Ao as the abundance of original sequences, any sequence with an R-value less than 0.5 was eliminated. Each reading was assigned a taxonomic attribution using the ecotag algorithm with the reference database of the EMBL repository, which included the sequences from several other databases [29]. The ecotag algorithm uses a phylogenetic structure approach to assign more reliable monophyletic units to the optimized sequences to ensure the return of molecular taxonomic units (MOTUs). Most diet analysis studies use the similarity limit of 97%, the default value for most grouping algorithms, for grouping on MOTUs [30]. This threshold was used because, on average, the minimum dissimilarity between the species for most target markers of metabarcoding primers, including COI, was approximately 3% [31]. Metagenomics were done with permission given by CGEN number A8E3D94.

### Insect identification and classification

A list of items in the diet of each bat species studied here was generated concerning the compatibility of the identity index returned by the ecotag algorithm to the highest possible taxonomic level to be estimated, with a minimum of 97% for species, 95% for genus, 90% for family, and 85% for order [32]. We used arthropods feeding behavior and lifestyle, and the scientific and technical literature [33] to classify insect species into functional groups. We considered the species considered agricultural pests in the Brazilian agroecosystems to be “pest insects.” “Predators” comprised of insect species known to prey upon other insects as some species could change their diet according to their life stage. For example, Chrysopidae are the only predators feeding on pollen as larvae and nectar as adults. Consequently, we only considered the pests that were predatory at one stage of their life cycle and could not harm plants in the other life stages to be predators. However, we made an exception for one species of the Syrphidae because this family is known to be a pollinator of several plant species. The species that benefited from human-caused ecological conditions, such as urbanization, were classified as “synanthropic.” The species known to pollinate wild plants or crops were classified as “pollinators.” All other species were classified as “other”.

### Valuating ecosystem services

To estimate the ecosystem service of biological control provided we considered the predation exerted by the bats on *Spodoptera frugiperda*, a polyphagous pest of great occurrence and high relevance, and that causes serious damage to several crops, especially maize, in Brazil [34, 35]. Also, this species was consumed by all the bat species in this study. Brazil is one of the world’s largest producers of corn, which is cultivated throughout the national territory and practically all year round. We made a conservative calculation of the economic value of the predation of a bat on *S*. *frugiperda*, as other factors, e.g., insecticide spraying, natural enemies, and climatic factors, may also affect the pest populations in the field.

First, we used previously published data to estimate the initial population of *S*. *frugiperda* in a 1-ha crop land [36], calculating the approximate abundance of *S*. *frugiperda* for several crops, including maize, in one harvest per season per year in the Brazilian Federal District from 2013 to 2017. Maize is mostly cultivated during the summer season in the Brazilian Cerrado, so that the critical damage inflicted by *S*. *frugiperda* was assumed to take place in November, December, and January, as reported by local farmers. We considered the mean number of moths collected by Fonseca-Medrano et al. [36] during maize harvests from 2013 to 2017 (Table 1) and divided it by the number of months related to the critical period of *S*. *frugiperda* (3 months) to obtain the number of moths per night per ha as the initial moth population (*N*_*i*_).

**Table 1:** The parameters used to quantify the ecosystem service of biological control provided by the bats preying on *Spodoptera frugiperda* in the maize field.

Parameter	Value	Reference
Mean moth (density/ha)	≈ 10	[71]
Value of a maize bag (R$)	84.43	[75]
Productivity of maize in Brazil (bags/ha)	≈ 101	[76]
Area cultivating maize in Brazil (Mha)	4,172	[76]
Productivity of maize in the Federal District (bags/ ha)	≈ 158	[76]
Area cultivating maize in the Federal District (ha)	21,800	[76]

Subsequently, we estimated that bat predation (*P*_*b*_) ranged from 1.5 (male bats) to 5 (pregnant female bats) moths/night/ha, similar to other studies [37]. We assumed a sex ratio of 1:1 on moth and bat populations and that each bat, independently of sex, preyed on two moths per night/ha to calculate the proportional reduction in the moth population (*i*.*e*., *P*_*b*_^*/*^*N*_*i*_). We chose to consider two moths per night to encompass males and females and to be more conservative. Also, many crop pests happen to be eared moths, and considering moths can difficulty bat predation [21], a conservative number is better. In Brazil, the commercial cultivation of maize is at 40,000 plants/ha (*M*_*d*_). The density of *S*. *frugiperda* caterpillars (*S*_*c*_) is usually one caterpillar per 10 plants in each hectare [38] because cannibalism occurs frequently among caterpillars, and only one caterpillar tends to remain on each plant [39]. Therefore, we calculated the reduction in the number of caterpillars/ha caused by bat predation (*R*_*bp*_) using the following formula:

$$R_{bp}=\left(\frac{M_{d}}{S_{c}}\right)\times \left(\frac{P_{b}}{N_{i}}\right)$$ (1)

We quantified the ecosystem services provided by bats by using a unit of maize productivity, maize bag (*VM*_*b*_) (60 kg/bag) [40] per hectare (*M*_*p*_) (or bags/ha), and the area of maize cultivation (*M*_*a*_) in Brazil and the Brazilian Federal District in the 2019/2020 harvest [41] as the main parameters (Table 1). The damage caused by *S*. *frugiperda* caterpillars ranges from 30 to 60% [42]. Thus, we used a threshold of damage (*D*_*c*_) of 30%, the maximum damage accepted by the local farmers, for the subsequent calculations. We then calculated the value of the ecosystem services provided by bats per hectare (*ES*_*b*_) during one maize harvest using the following formula:

$${ES}_{b}=\left[{VM}_{b}\times (\frac{D_{c}}{100})\times (\frac{P_{b}}{N_{i}})\right]\times ({VM}_{b}\times M_{a})$$ (2)

#### Statistical analysis

First, we assessed how the number of reads of MOTU in each insect family varied among the bat species by fitting a generalized linear model (GLM) with a Poisson distribution. The number of MOTUs was used as the response variable and bat species as the explanatory variable. The significance of the variables was assessed using the chi-square test [43]. The differences in the number of reads among the bat species were compared using the model contrast analysis. A residual analysis was performed to test the model’s fit [43].

We investigated the diversity of the diet of the bats by calculating the frequency of the insect families found in the feces of each bat species. We then compared the diversity of insect families consumed by each bat species by calculating the Shannon index of diversity (H’) and bootstrapped the data with 1000 randomizations to calculate the confidence intervals. The Shannon diversity index was used as a proxy for dietary diversity. We compared the Shannon index values using a modified t-test (Hutcheson t-test) with all the possible pairwise comparisons among the bat species [44]. A principal coordinate analysis analysis (PCoA) was used to verify the similarity in the composition of bat diets based on the insect families consumed. We used the Morisita index of similarity in our analysis, followed by a permutational analysis of variance (PERMANOVA) to test the significance of the groups and possible differences int terms of insect families composition in the diet of each bat species [44].

We fitted a GLM with a quasi-Poisson distribution for under-dispersed data to assess which functional group was more preyed on by the bats, regardless of the bat species. We used the number of MOTU reads per bat species as the response variable and the insect functional group as an explanatory variable in the model. We investigated whether each bat species preyed on any specific functional group by fitting a GLM with binomial distribution or quasibinomial distribution for the under-dispersed data. The proportion of MOTU reads for each functional group was used as the response variable and the functional groups’ identity as the explanatory variable. The analysis was performed separately for each bat species. As insect pests were found in the feces of all the bats, we used a PCoA to verify whether insectivorous bats preyed on different pest insect species (urban and agricultural), as described above. The GLMs were fitted using the software R [38] and all the other analyses were performed using the software PAST [44].

## Results

### Diet composition and diversity

We analyzed the fecal samples of 175 bats of five species captured in shelters in buildings in three cities in the Federal District of Brazil (see Fig 1). Only 43 fecal samples reached the sequencing stage: 15 *N*. *laticaudatus*, 7 *C*. *planirostris*, 11 *H*. *diaphanopterus*, 4 *M*. *molossus* and 6 *E*. *perotis*. These bats were found to consume arthropods belonging to 10 orders, 61 families, 39 genera, and 83 morphospecies. We identified 40 insect species, 21 at the genus level and 19 at the species level. We also identified one spider mite (Acari: Tetranychidae) species (Table 2). Among the taxa associated with arthropods, 18.5% of MOTUs are classified as pests, 1.1% as pollinators, while the other functional groups do not reach 1%. Pests represent 8.9% of all replicas found in bat guano (Table 2).

**Table 2:** The DNA samples of the arthropods found in the feces of insectivorous bats in the city roosts and colonies in the Federal District of Brazil with the classification of the arthropods into functional groups, number of MOTUs, replicates and identities given by the GenBank.

Class/Order/Family/Species	Hd	Ep	Nl	Mn	Cp	Functional group	MOTUs	Replicates	Identity	Observation
**Acari**										
** Trombidiformes**										
Tetranychidae										
*Tetranychus ludeni*		1				Pest	1	40	0.993	
**Insecta**										
** Isoptera**										
Rhinotermitidae										
*Rhinotermitidae* sp.1	1									
Termitidae										
*Termitidae* sp.1			1							
**Coleoptera**	1	1								
Carabidae										
Carabidae sp.1			1	1						
*Lecanomerus* sp.					1	Predator	8	2257	0.961	
Chrysomelidae										
Chrysomelidae sp.1					1					
Curculionidae										
Curculionidae sp.1			1							
Dytiscidae										
Dytiscidae sp.1					1					
Geotrupidae										
Geotrupidae sp.1					1					
Gyrinidae										
Gyrinidae sp.1			1	1	1					
*Porrorhynchus* sp.					1	Other	3	260	0.951	
Scarabaeidae										
Scarabaeidae sp.1			1	1						
Staphylinidae										
Staphylinidae sp.1			1							
Tenebrionidae										
Tenebrionidae sp.1					1					
**Diptera**										
Calliphoridae										
Calliphoridae sp.1		1								
*Chrysomya megacephala*					1	Synathropic	1	25	1.000	
Cecidomyiidae										
Cecidomyiidae sp.1					1					
Chironomidae										
Chironomidae sp.1			1	1						
Culicidae										
Culicidae sp.1		1	1							
*Culex declarator*				1		Synanthropic	1	6	1.000	
Drosophilidae										
*Drosophila* sp.			1	1		Synanthropic	4	111	0.951	
*Drosophila nasuta*	1		1	1		Synanthropic	4	1697	0.997	
Limoniidae										
*Rhipidia domestica*				1		Other	6	13	0.997	
Mycetophilidae										
Mycetophilidae sp.1				1						
Sarcophagidae										
*Amobia* sp.			1							
Syrphidae										
Syrphidae sp.1	1		1	1						
*Ocyptamus* sp.					1	Pollinator	3	526	0.951	
Tephritidae										
Tephritidae sp.1			1							
*Eurosta* sp.					1	Other	1	63	0.954	
**Hemiptera**	1	1								
Alydidae										
*Neomegalotomus parvus*			1	1	1	Pest	159	11772	0.997	
Aphididae										
*Rhopalosiphum padi*			1			Pest	1	132	1.000	cf
Cicadidae										
Cicadidae sp.1		1								
Cercopidae										
*Mahanarva* sp.	1					Pest	1	17	0.961	
Delphacidae										
*Chionomus* sp.			1		1	Other	2	714	0.967	
Lygaeidae										
Lygaeidae sp.1			1	1	1					
Membracidae										
Membracidae sp.1					1					
Miridae										
*Campylloma* sp.			1	1		Predator	1	10	1.000	
Nabidae										
Nabidae sp.1		1	1	1						
Pentatomidae										
Pentatomidae sp.1					1					
Pyrrhocoridae										
*Dysdercus* sp.			1			Pest	6	8027	0.961	
**Hymenoptera**										
Apidae										
*Apis mellifera*	1					Pollinator	1	2	1.000	Exotic
*Eucera* sp.					1	Pollinator	1	82	0.967	
Braconidae										
*Notiospathius* sp.	1					Parasitoid	1	16	1.000	
Eucharitidae										
Eucharitidae sp.1	1									
Formicidae										
Formicidae sp.1	1		1		1					
Vespidae										
*Agelaia pallipes*			1		1	Predator	13	8405	0.997	
**Lepidoptera**										
Batrachedridae										
*Batrachedra* sp.	1					Pest	1	2	0.957	
Crambidae										
*Diatraea saccharalis*	1					Pest	1	18	1.000	
*Pyrausta panopealis*		1				Pest	1	226	0.970	cf
*Spoladea recurvalis*			1			Pest	2	230	0.997	
Gelechiidae										
Gelechiidae sp.1	1									
Geometridae										
Geometridae sp.1				1						
*Cyclophora* sp.			1			Pest	1	15	0.988	
Gracillariidae										
Gracillariidae sp.1			1							
Hesperiidae										
Hesperiidae sp.1	1	1								
Lycaenidae										
Lycaenidae sp.1			1	1						
Noctuidae										
Noctuidae sp.1		1								
*Eudocima* sp.		1				Pest	27	11155	0.957	
*Elaphria agrotina*	1			1		Pest	54	1346	0.997	
*Feltia jaculifera*	1					Pest	1	943	0.974	cf
*Helicoverpa zea*	1	1				Pest	3	220	0.997	
*Heliothis* sp.		1				Pest	1	81	0.961	
*Spodoptera* sp.				1		Pest	10	56	0.997	
*Spodoptera frugiperda*	1	1	1	1	1	Pest	147	103575	0.997	
Nolidae										
*Gabala* sp.			1				1	228	0.951	
Nymphalidae						Pest				
Nymphalidae sp.1	1	1		1	1					
*Junonia* sp.			1			Pollinator	3	241	0.951	
*Parthenos* sp.		1				Pollinator	11	1377	0.951	
Oecophoridae										
Oecophoridae sp.1			1							
Papilionidae										
*Archon* sp.			1	1		Pollinator	5	1466	0.964	
*Protesilaus* sp.		1				Pollinator	4	781	0.954	
Plutellidae										
*Plutella xylostella*			1			Pest	4	1865	0.990	
Pyralidae										
*Elasmopalpus lignosellus*			1			Pest	50	2823	0.991	
Sesiidae										
Sesiidae sp.1		1		1						
Sphingidae										
Sphingidae sp.1		1								
**Mantodea**										
Hymenopodidae										
Hymenopodidae sp.1	1									
Liturgusidae										
Liturgusidae sp.1	1		1							
Mantidae										
Mantidae sp.1	1		1							
**Neuroptera**										
Chrysopidae										
Chrysopidae sp.1	1									
*Chrysoperla externa*			1			Predator	1	2	1.000	
Mantispidae										
Mantispidae sp.1			1							
**Orthoptera**										
Rhaphidophoridae										
Rhaphidophoridae sp.1			1							
Tettigoniidae										
Tettigoniidae sp.1	1									

Cf = closest taxonomic identification given by the GenBank.

The observations refer to the possible new records of exotic species DNA retrieved from the bat feces samples. Hd, *Histiotus diaphanopterus*; Ep, *Eumops perotis;* Nl, *Nyctinomops laticaudatus;* Mm, *Molossus molossus;* Cp, *Cynomops planirostris*.

The five species of bats were found to prey on nine insect orders, of which Lepidoptera was most frequently consumed at 33.87%, followed by Diptera at 17.7%, Hemiptera at 16.9%, and Coleoptera at 13.71% (Table 2). Lepidopteran insects were mostly consumed by *Eumops perotis* at 64.7%, followed by *H*.*istiotus diaphanopterus* at 36.0%, *Nyctinomops laticaudatus* at 31.6%, and *Molossus molossus* at 31.8%. On the other hand, at 31.8%, Coleopterans were the most common food item in the diet of *Cynomops planirostris*. The insects of the order Isoptera were hunted only by *H*. *diaphanopterus* and *M*. *molossus*, whereas those of Mantodea, Neuroptera, and Orthoptera were consumed only by *H*. *diaphanopterus* and *N*. *laticaudatus*. Only *E*. *perotis* was found prey on the arthropods of the subclass Acari. *Nyctinomops laticaudatus* was found to consume the largest number of food items (n = 38) and insect families (n = 36).

**Fig 2:**
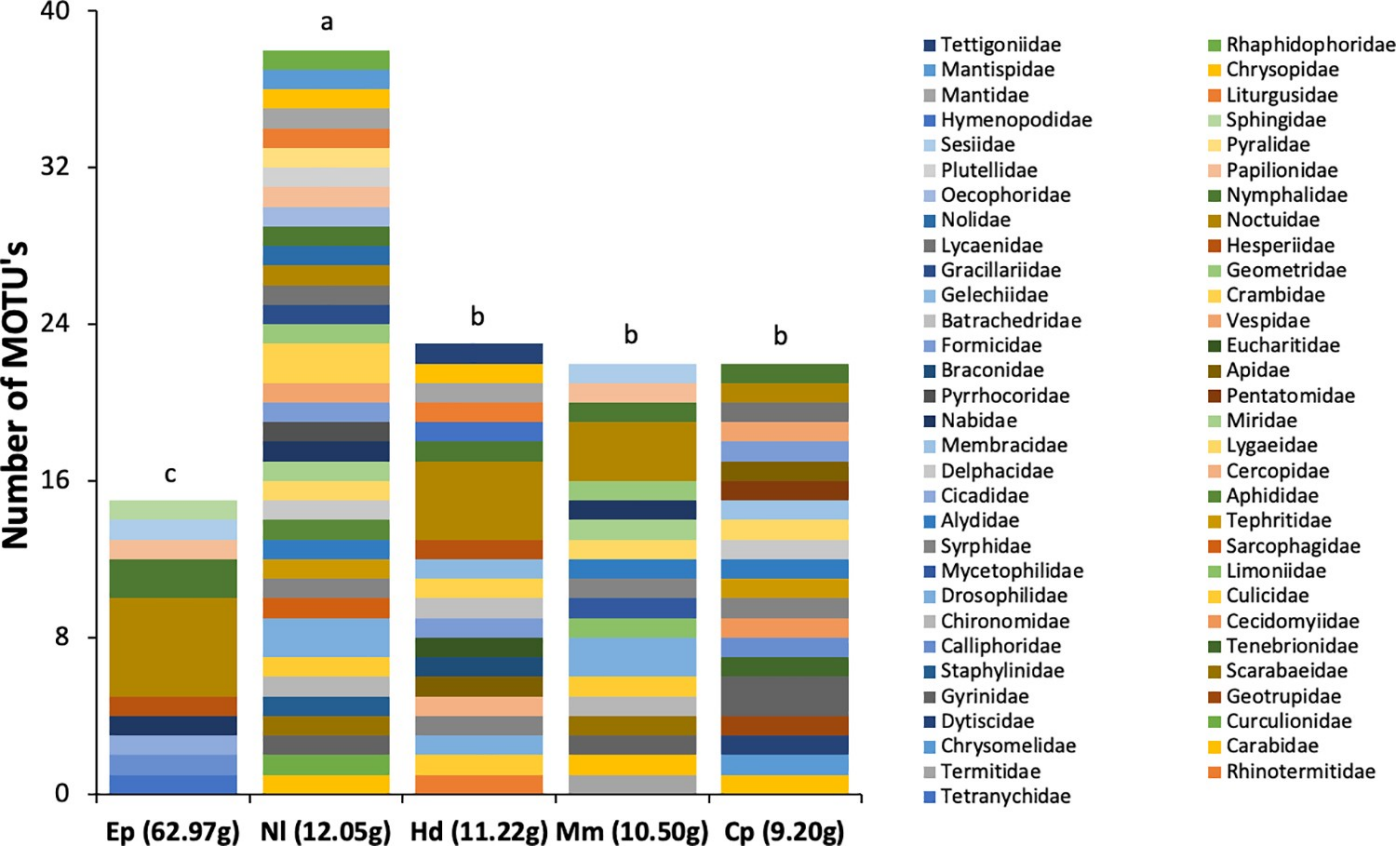
The number of reads of molecular taxonomic units (MOTU’s) of the insect families found in the feces samples of the insectivore bats *Eumops perotis* (Ep), *Nyctinomops laticaudatus* (Nl), *Histiotus diaphanopterus* (Hd), *Molossus molossus* (Mm), and *Cynomops planirostris* (Cp) found roosting in buildings in the cities of the Federal District of Brazil. Species were organized in the chart according to their body mass (in parenthesis). Different lower-case letters above the bars indicate significant differences by model contrast analysis (p < 0.05).

The insect species belonging to Nymphalidae (Lepidoptera), Gyrinidae (Coleoptera), Lygaeidae (Hemiptera), and Nabidae (Hemiptera) as well as *Spodoptera frugiperda* (Lepidoptera: Noctuidae) were consumed by all the bat species (Table 2 and Fig 2). Four of the agricultural pest insects identified in the feces are not found in Brazil (Table 2) suggesting the bats probably were preying on species phylogenetically related to these foreign species. The number of reads of MOTUs from insect families significantly differed among the bat species (χ^^2^^ = 11.21, df = 4, p = 0.024). The highest number of MOTUs was found in the feces of *N*. *laticaudatus* and the lowest in the feces of *E*. *perotis*, while the other species had similar, intermediate values (Fig 2).

**Fig 3:**
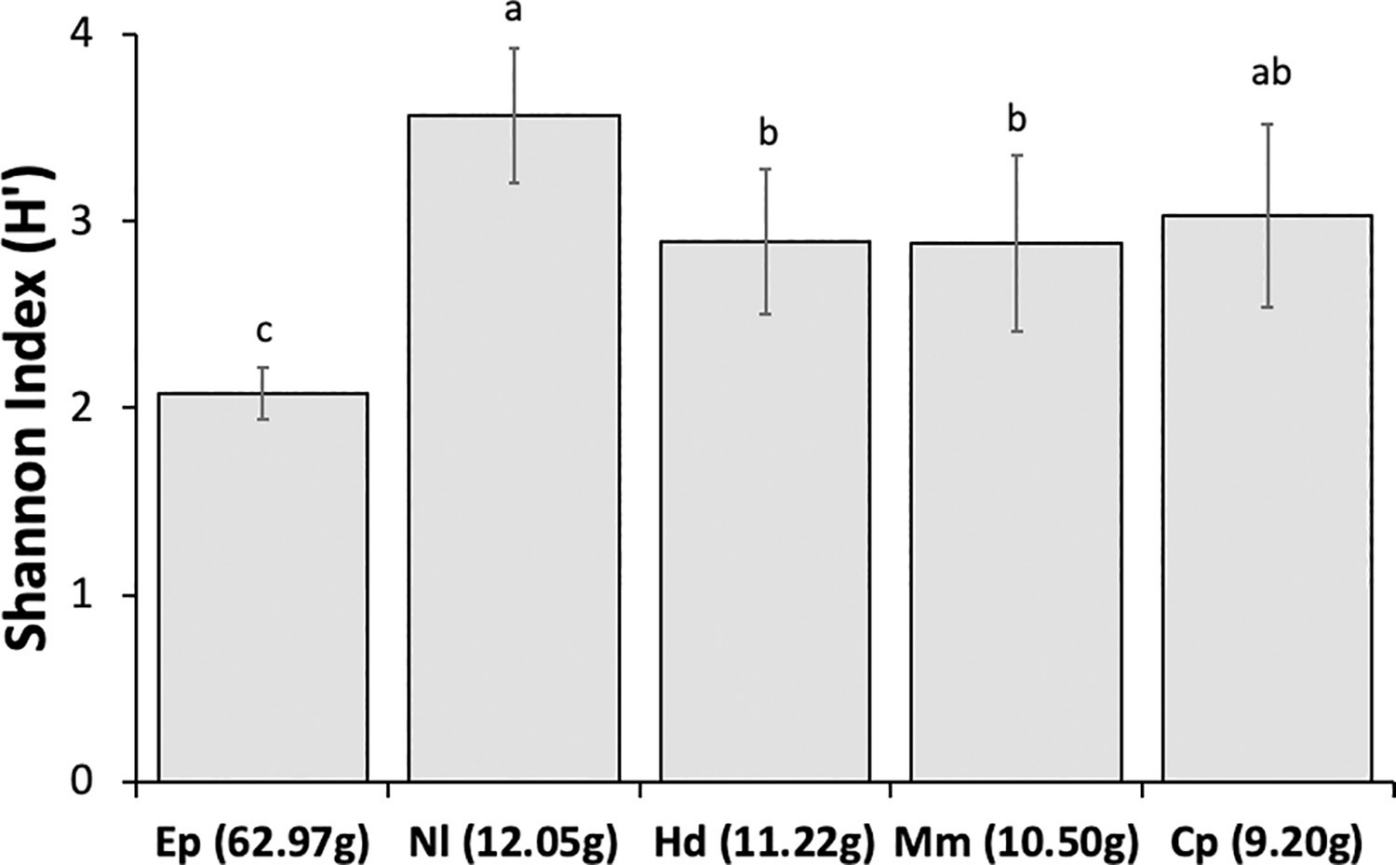
Shannon index (H′) of the diversity (± confidence interval bars) of insect families as food prey items in the diet of the insectivorous bats *Eumops perotis* (Ep), *Nyctinomops laticaudatus* (Nl), *Histiotus diaphanopterus* (Hd), *Molossus molossus* (Mm), and *Cynomops planirostris* (Cp) in city roosts and colonies in the Federal District of Brazil. Species were organized in the chart according to their body mass (in parenthesis). Different lower-case letters above the bars indicate significant differences after all possible pairwise comparisons among bat species using the Hutcheson’s t-test (P < 0.05).

**Fig 4:**
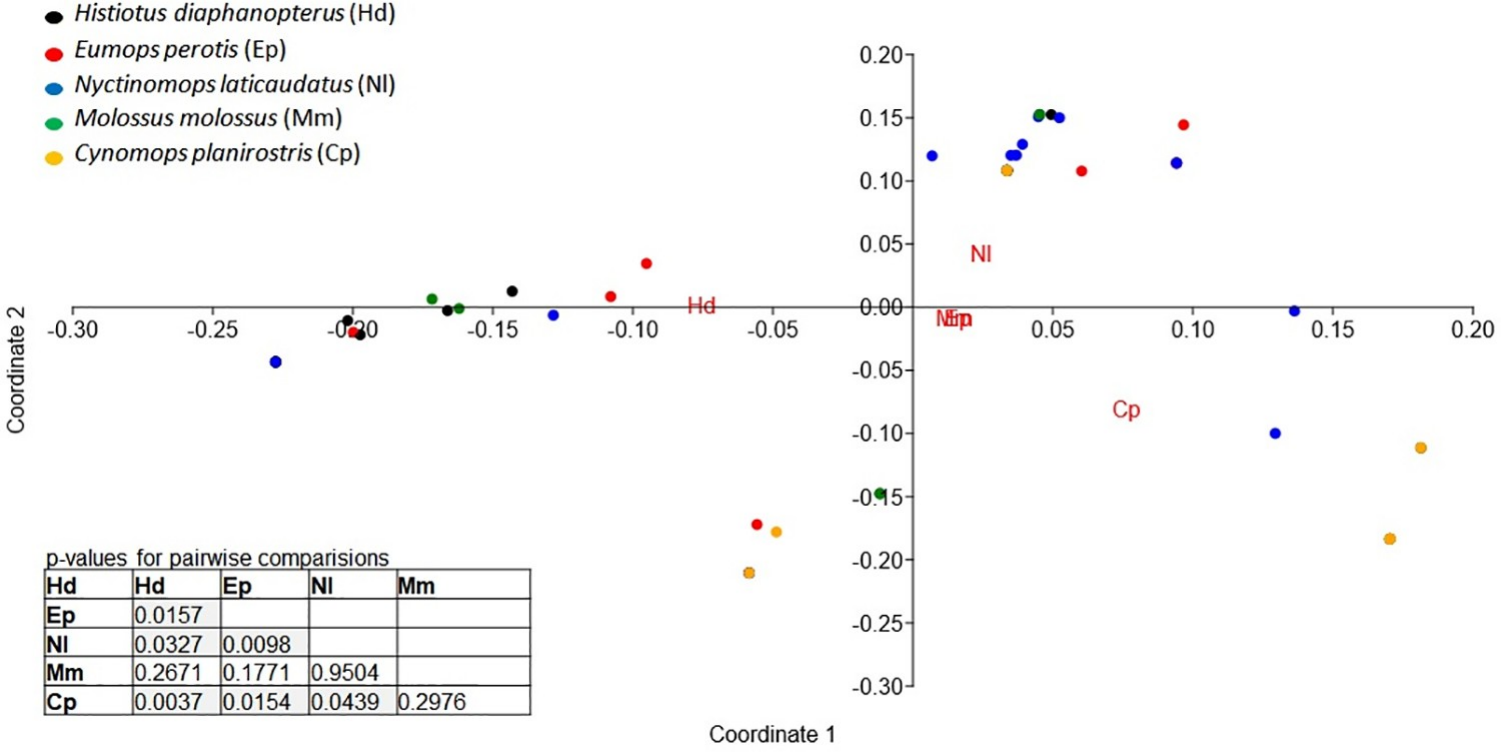
Principal coordinate analysis (PCoA) based on the similarity (Morisita index) of DNA samples of different insect families obtained in the feces of the insectivorous bats *Eumops perotis* (Ep), *Nyctinomops laticaudatus* (Nl), *Histiotus diaphanopterus* (Hd), *Molossus molossus* (Mm), and *Cynomops planirostris* (Cp) roosting in cities buildings in the Federal District of Brazil, showing the p-values for all pairwise comparisons among bat species.

Following the same trend, the diversity of the diet of *N*. *laticaudatus* (H′ = 3.565) was significantly higher than that of other bat species, and the diet of *E*. *perotis* (H′ = 2.079) was the least diverse. The diets of *C*. *planirostris* (H′ = 3.028), *H*. *diaphanopterus* (H′ = 2.894), and *M*. *molossus* (H′ = 2.878) exhibited similar, intermediate diversity (Fig 3). The diet composition of the five bat species differed remarkably among themselves (Fig 4) (Pseudo F = 2.264, p = 0.0001). The diet composition of *H*. *diaphanopterus* and *E*. *perotis*, mainly consisted of insects from the Noctuidae family and did not differ significantly. The bat *N*. *laticaudatus* presented the most diverse diet among the species evaluated, followed by *M*. *molossus*, which preyed on insect of various families. In contrast, C. *planirostris* presented the least diverse and most exclusive diet composition among the bat species (Fig 4).

#### Ecosystem services provided by bats

**Fig 5:**
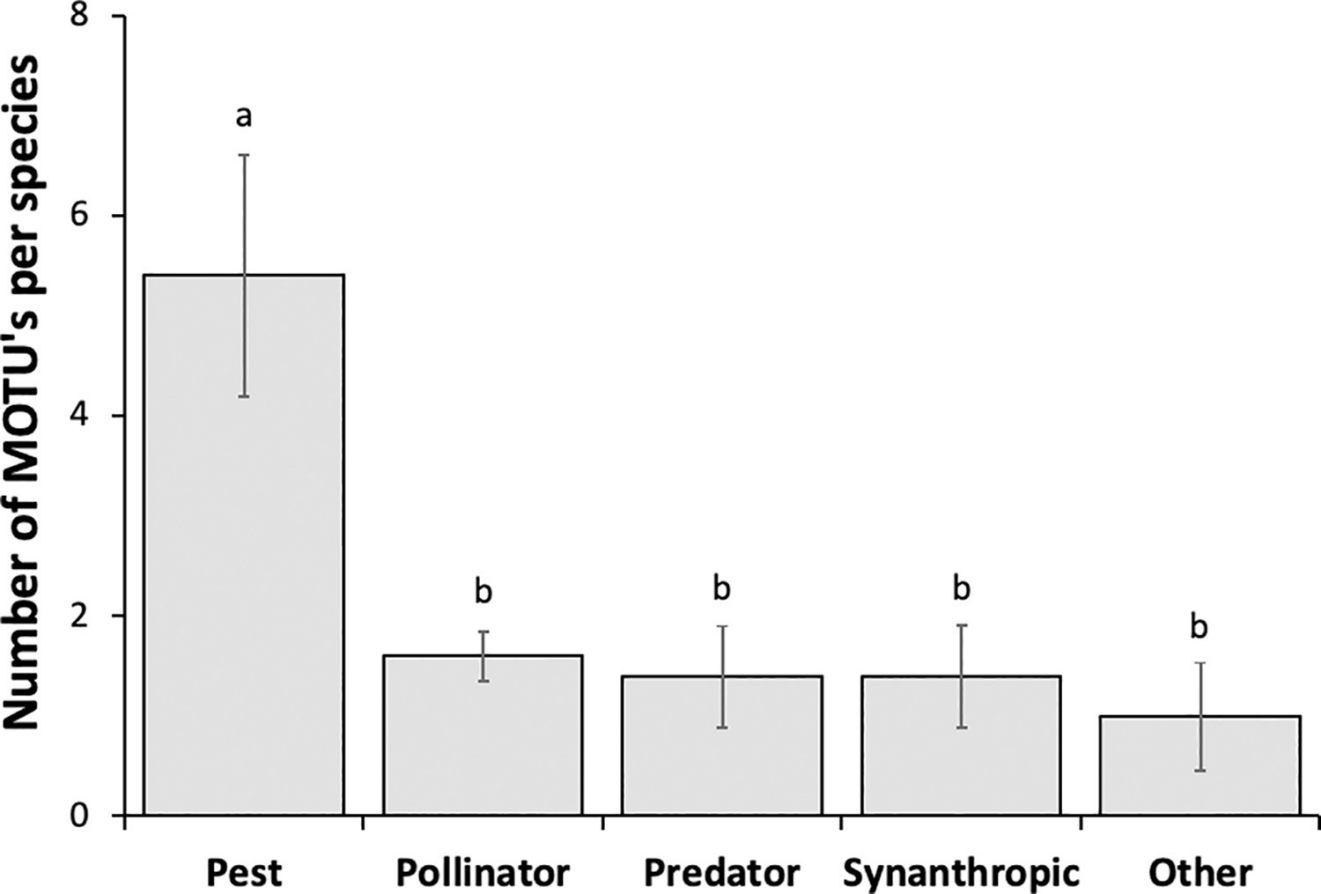
Number of the reads of molecular taxonomic units (MOTU’s) of DNA samples per bat species based on insect DNA samples classified in different functional groups and present in the feces of the insectivorous bats roosting in buildings in the cities of the Federal District of Brazil. Different lower-case letters above the bars indicate significant differences by model contrast analysis (p < 0.05).

**Fig 6:**
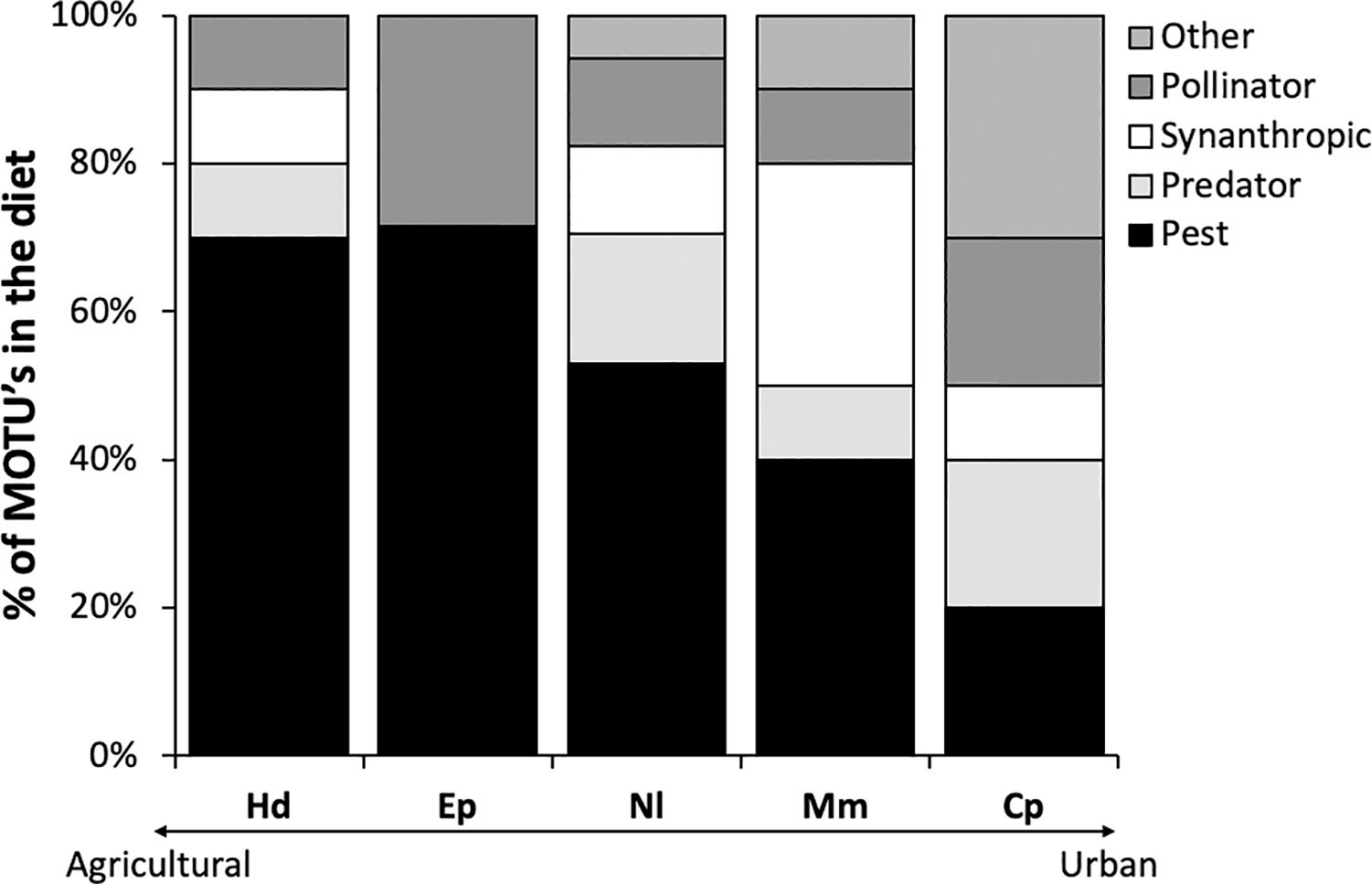
Percentage of the reads of molecular taxonomic units (MOTU’s) of DNA samples based on insect DNA samples classified in different functional groups and present in the feces of the insectivorous bats *Histiotus diaphanopterus* (Hd), *Eumops perotis* (Ep), *Nyctinomops laticaudatus* (Nl), *Molossus molossus* (Mm), and *Cynomops planirostris* (Cp) roosting in buildings in the cities of the Federal District of Brazil. Species were organized in the chart according to the landscape matrix were their roosts or colonies were sampled.

We verified that, in the feces of the five bat species, a significantly higher number of MOTUs corresponded to those of agricultural pests than to those of other insect functional groups (χ^^2^^ = 25.36, df = 4, p < 0.001) (Fig 5). While all the bat species hunted pest insects, only *E*. *perotis* did not prey upon synanthropic species (Fig 6). We found that 70% of the MOTUs detected in *H*. *diaphanopterus* feces were from pest insects, different from the number of MOTUs from other functional groups of insects (χ^^2^^ = 13.26, df = 3, p = 0.004). *Eumops perotis* fed on only pest (71.43%) and pollinator species (28.57%); there were no significant differences between the proportion of MOTUs for these two functional groups (χ^^2^^ = 2.57, df = 1, p = 0.103) (Fig 6). *N*. *laticaudatus* preyed on all functional groups of insects but significantly more on pest species (59.94%) (χ^^2^^ = 13.48, df = 4, p = 0.009). Although *M*. *molossus* also hunted all functional groups of insects, the proportion of MOTUs detected in its feces was similar among all functional groups (χ^^2^^ = 4.86, df = 4, p = 0.302). Following the same pattern, *C*. *planirostris* fed on a similar proportion of all functional groups of insects (χ^^2^^ = 1.30, df = 4, p = 0.862) (Fig 6).

**Fig 7:**
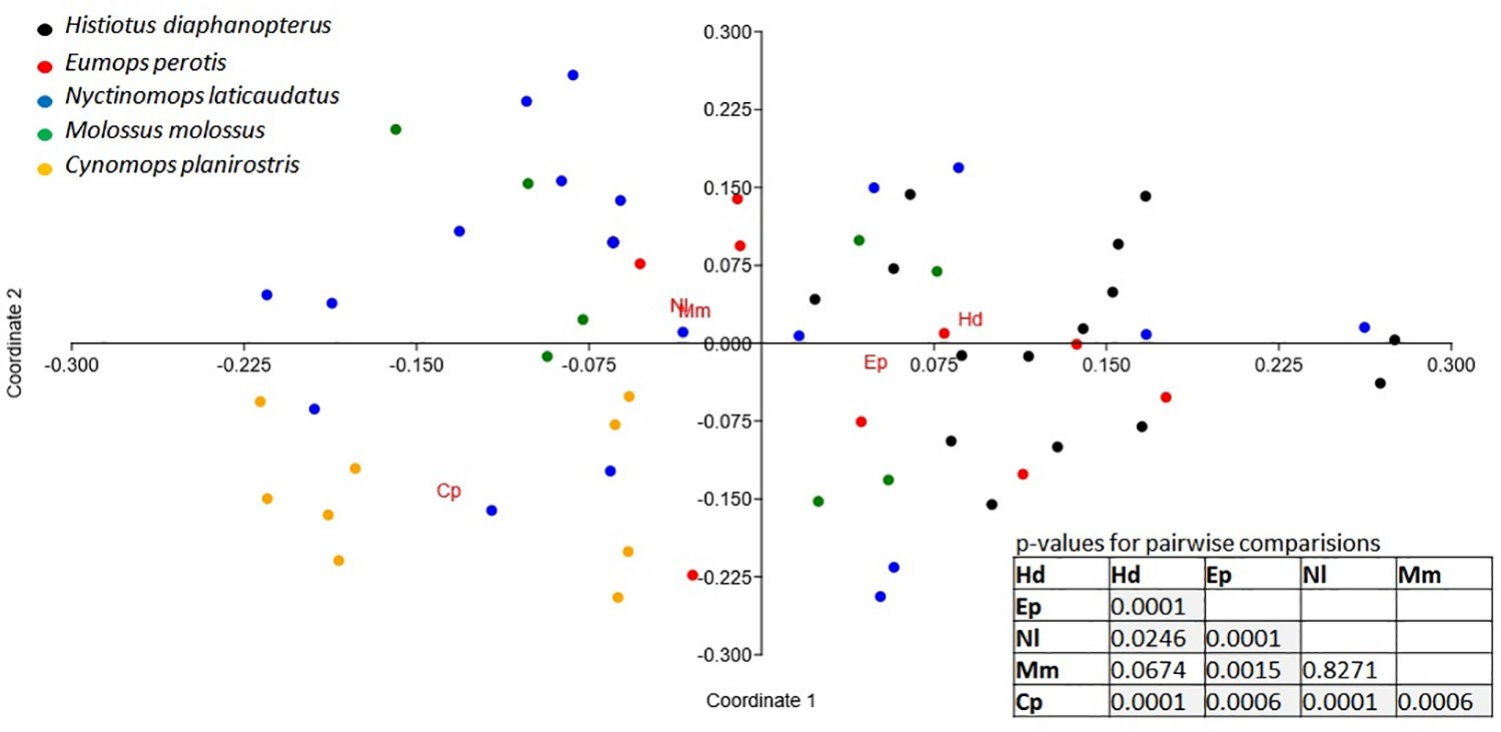
Principal coordinate analysis (PCoA) based on the similarity (Morisita index) of DNA samples of different insect species classified into agricultural or urban pests obtained in the feces of the insectivorous bats *Eumops perotis* (Ep), *Nyctinomops laticaudatus* (Nl), *Histiotus diaphanopterus* (Hd), *Molossus molossus* (Mm), and *Cynomops planirostris* (Cp) in city roosts and colonies in the Federal District of Brazil, showing the p-values for all pairwise comparisons among bat species.

Considering only the pest insects consumed in agricultural and urban areas, different bat species feed on different species of insects (Fig 7). The bat *H*. *diaphanopterus* consumed mostly agricultural pests and was very distant from other bat species. The group of *M*. *molossus*, *E*. *perotis* and *C*. *planirostris* hunted urban/synanthropic insect species with a low difference in the species they consumed. This group was closer to *N*. *laticaudatus*, which fed on a mixed diet of several functional groups. However, *N*. *laticaudatus* preyed more on pest insects than the urban pests (Fig 7).

### Predation value of bats

Assuming the initial population of *S*. *frugiperda* moths was approximately 10 moths per hectare (ha) (Table 1), we calculated that one bat consuming two moths per night could reduce the S. *frugiperda* population by 20%. The maize crop is infested with one caterpillar for every 10 plants, generating a population of 4,000 caterpillars per ha [36]. In this way, the 5 female moths flying each night can generate a population of 4,000 caterpillars per ha. Considering a reduction of 20%, the population would be 3,200 caterpillars per ha. The damage caused by *S*. *frugiperda* caterpillars in the crop can vary from 20 to 100%. Because the farmers may use several management strategies, we considered a loss of 30%. Thus, with bats reducing the moth population to 3,200 caterpillars, the loss would be 24% (see formula 1). Therefore, bats could save approximately US$ 1 per maize bag, according to the current price of one maize bag in Brazil (Table 1). Considering maize productivity in Brazil and the area cultivating maize in the country (Table 1), bats can save approximately US$ 94.00 per ha. The value of *S*. *frugiperda* predation by bats is estimated at US$ 390.6 million per harvest in Brazil (see formula 2). In the Federal District, where maize productivity is higher than the overall maize productivity of Brazil (Table 1), the value of bat predation can reach US$ 3.19 million per harvest.

## Discussion

Here, we showed that bats inhabiting cities prey on several arthropod species relevant to urban and agricultural environments. Even in cities, bats consumed mostly insects that were agricultural pests. Thus, we have confirmed our hypothesis that, despite inhabiting cities, bats continue to play an essential role in providing biological control suppressing agricultural pests. The pest suppression by bats is an ecosystem service little-recognized worldwide [19] especially in Brazil, where bats are recognized only as rabies vectors [1]. In addition, we have demonstrated a complementary effect on the predation of insects among the studied bat species due to their diets of different pests and synanthropic arthropods. Also, we tested whether insectivorous bats attacked mainly synanthropic species, such as flies and mosquitoes carrying human diseases and causing outbreaks when their populations in human habitats became overabundant. However, this hypothesis was not confirmed.

### The consumption of different food items by bats

Regardless of the bat species, the bats’ high consumption of different food items was likely due to two factors. First, the bat species were insectivorous and consumed a broad range of invertebrates [45]. Bats may consume the equivalent to 80%–100% of their body mass, depending on insect order availability throughout time [2, 46]. The second factor is related to the abundance and availability of the resources, i.e., insects, in or near urban areas. Insects are the most abundant and diverse organisms on Earth, with approximately one million described and at least 5 million undiscovered species [47]. The highest biodiversity of insects in the world can be found in Brazil, a megadiverse country inhabiting approximately 9% of the world’s total insect species [47].

Among the orders of the consumed insects (Table 2), Coleoptera, Diptera, Hymenoptera, and Lepidoptera are megadiverse orders comprising more than 70% of the total known insect species. Notably, the activity schedules of many of these insect species coincide with the nocturnal foraging behavior of bats [48]. For example, the families Noctuidae, Crambidae, Scarabaeidae, and Curculionidae mainly have nocturnal foraging habits [49, 50]. The insectivorous bats adjust their nightly activities to match the availability of their prey, maximizing foraging success and energy gains [51, 52].

Some species, mainly of the Lepidoptera order, such as *Diatraea saccharalis* (Crambidae), *Elaphria agrotina* (Noctuidae), *Helicoverpa zea* (Noctuidae), *Spodoptera sp*. (Noctuidae), *S*. *frugiperda* (Noctuidae), *Plutella xylostella* (Plutellidae), and *Elasmopalpus lignosellus* (Pyralidae), are considered polyphagous pests that feed on diverse economically important small and large-scale crops, including soybeans, cotton, sorghum, corn, sunflower, sugar cane, peanuts, beans, and tomatoes [53, 54]. These results reinforce the importance of bats as biological control agents of important agricultural pests and providers of crop-related ecosystem services.

In addition, we have shown for the first time that bats consume soybean pests, such as *Neomegalotomus parvus*, *S*. *eridania*, and *E*. *lignosellus*, sugarcane borer *D*. *saccharalis*, and cotton pests such as *Dysdercus* sp. and *Heliothis* sp. Corn pests, such as *H*. *zea* and *S*. *frugiperda*, have already been registered as bat food items in the USA [55, 56]. Among the insects identified by DNA metabarcoding, four agricultural pest insect species, *Rhopalosiphum padi* (Hemiptera: Aphididae), *Pyrausta panopealis* (Lepidoptera: Crambidae), *Feltia jaculifera* (Lepidoptera: Noctuidae), and *Gabala* sp. (Lepidoptera: Nolidae), have not been documented in Brazil. Although this is a fascinating result, it needs to be validated carefully.

The environmental DNA (eDNA) metabarcoding has been used to assess the biodiversity of several taxa in multiple ecosystems in different parts of the world. In many cases, they could detect the species missed by the traditional approach [57, 58]. Although metabarcoding methods have been under intensive development over the past 10 years, several gaps remain. Zenker et al. [58] found that the insect diversity results underrepresented the true magnitude of insect diversity expected from the samples obtained with automatic light traps in Brazil, likely due to the storage of eDNA samples under suboptimal conditions. Thus, we recognized limitations in our method and assumed that taxonomic misidentification might have occurred mainly because we used bat fecal material. Another aspect that should be considered is that some insects, such as aphids, might not have been directly targeted by bats. We presume they were preyed upon by another predator insect consumed by bats, and so they might be detected by metabarcoding [59].

The role of bats or any general predator in suppressing prey populations depends on their ability to track and exploit the available prey [60]. Although the consumption of synanthropic insects was lower than that of the other groups, we found other prey associated with human habitats, such as *Culex* and *Chrysomya* mosquitoes. *Culex declarator* is the primary vector of St. Louis encephalitis and other arboviruses [61]. In Brazil, densely populated cities are infested by *Culex* and *Aedes* mosquitoes. Ecological changes, such as deforestation due to human settlements, can affect virus transmission cycles. In recent years, Brazil has presented higher rates of diseases, such as dengue, Zika, and chikungunya, accounting for approximately 70% of the reported dengue fever cases in the Americas [61–63]. Thus, the consumption of vector insects, such as *C*. *declarator* and *Chrysomya megacephala*, which are the vectors of enteric bacteria and protozoa associated with arbovirus transmission and secondary myiasis in several animal species [61, 64] can help to reduce the risk of infection in humans and animals in urban areas. While *C*. *declarator* is a nocturnal species [61], some vectors, such as *A*. *aegypti*, were not found in the bat diet, probably because of the difference in the time of activity between bats and this daytime vector [65].

Our results showed that the functional group of agricultural pests was consumed three times more than any other group by bats in the cities. Among the bat species we evaluated, we observed that the consumption of agricultural pests represented more than half of the insects consumed by a bat species, except for *M*. *molossus* and *C*. *planirostris* that had a more diversified diet (Fig 5). Furthermore, *M*. *molossus* consumed the highest number of insects from the synanthropic insect group, whereas *E*. *perotis* did not consume any insects from this group (Fig 5). As a result, the consumption of different functional groups of insects by the bats provides multiple and complementary ecosystem services beyond the city limits.

### The use of DNA metabarcoding

A growing body of literature highlights the ecosystem services provided by insectivorous bats in agricultural landscapes [3, 66–69]. However, the studies that evaluated the food items consumed by bats were able to identify the insects up to the order level before 2010 [64] and to the species level only after 2010 [24, 70, 71], mainly due to the difficulty in identifying insects from the small pieces found in bat feces. In this study, we identified several insects up to the family level and others up to the species level using DNA metabarcoding, which required smaller quantities of materials than the traditional taxonomic tools. As a result, we could identify the insect species consumed by bats more precisely. The DNA metabarcoding analysis, unlike traditional taxonomic tools, uses small quantities of materials.

Of the five bat species analyzed here, there is only data on the diet of *M*. *molossus* and *E*. *perotis* collected from the urban areas of Colombia [72] and Brazil [73], respectively. In both studies, the feces of the bat species were checked with a stereomicroscope and found to have an abundance of food items related to Coleoptera. As found for other species, this result may reflect the method used to evaluate the diet. Our metabarcoding analysis indicated that the bat species preyed more heavily on Lepidoptera than on Coleoptera.

However, the DNA method can also affect the detection of Coleoptera considering there is a universal and blocking primer mismatches limiting e-DNA metabarcoding analysis [74], as we have discussed above. Thus, the identification of Coleoptera is not easy as it is the visual identification of the hard tegument. Lastly, this study is the first report of the items consumed by *H*. *diaphanopterus*, a recently described bat species that preys on Lepidoptera and other insect orders such as Mantodea, Diptera, and Hymenoptera.

### Bats travel a long distance to forage

The presence of many agriculture pests in the feces of the bats may be because the bats fly to the agricultural areas to feed. Although they roost in the city, the competition for food is probably too high due to the decreasing insect biomass in urban areas [75, 76]. Alternatively, it may not be too far for the bats to fly to the nearby rural area [77]. On the other hand, herbivorous arthropod pests are often abundant in urban areas, and urban warming may cause outbreaks of these pests [78]. Since the agricultural areas and cities are close together in the Federal District, many insects may be dispersed into agricultural areas, especially when the population density of the urban insects becomes too high. Thus, the factors affecting insect consumption by bats are resource availability, foraging time compatibility, and proximity between the roosting and foraging areas.

### The economics of insect predation by bats

Regardless of the origin of the pests, we were able to estimate the value of bat predation at US$ 390.6 million per harvest in the Brazilian territory. The predation value we have calculated for *S*. *frugiperda* on maize in different scenarios only represents the indirect effects of bat predation when the adult moths are removed. These predation values have not accounted for when the cost of pest population growth is considered because the ecosystem service by the bats to Brazilian agriculture is almost invisible. In other words, the US$ 17 billion expenditure for pest suppression in Brazil [79] and the damage inflicted by several economically representative species, such as those found here, could be even greater if bats were absent in the distinct ecosystems, especially in the Cerrado biome, in the country. For the first time in Brazil, we quantified this critical ecosystem service provided by the bats and demonstrated the relevant role of bats in the Brazilian economy largely based on the export of agricultural commodities. It is noteworthy that such values must be interpreted with caution. Our results present a very conservative estimate and not an absolute value for the ecosystem services provided by bats. In addition, we considered an ideal and best-case scenario of bat species constantly occurring in all corn production areas in Brazil. However, even if one or another species studied here does not occur widely in the country, the same service can be performed by other species of insectivorous bats, especially in a megadiverse country like Brazil.

### The necessity of bat conservation

The bat species analyzed here were found to differ in their diet of insect species, composed mainly of daytime and night-time agricultural pests. The smallest bat species, *C*. *planirostris*, preyed on pests the least. However, its diet contains a pest of soybean, which is cultivated around the city of Brasília. This result reinforces that the conservation of biodiversity is related to the provision of ecosystem services. The pool of species complements each other, resulting in the simultaneous regulation of more pests and other insects.

The results presented here are significant because bats are persecuted in the agricultural lands of Latin America because of their role in rabies transmission [1]. These results are sufficient to call attention to the necessity of conserving bats as one of the major players in pest management. The consumption and reduction of synanthropic insects by bats can help improve people’s perception of bats.

In addition, the use of pesticides is expensive, and resistant insects can be developed, leading to the loss or reduction of pest control [80]. In recent years, Brazil has become the largest consumer of pesticides (51% used in soybean crops) with an annual US$ 17 billion expenditure for pest control [79]. The conservation of bats may help reduce the cost of pesticides while increasing the effectiveness of pest suppression.

Since the 1950s, anthropogenic environmental changes have intensified in the Cerrado biome, which is considered Brazil’s last agricultural frontier, where agricultural expansion is the predominant cause of species and habitat loss [81]. Thus, best agriculture practices are needed to maintain the ecosystem services provided by bats, even with them inhabiting cities and consuming crop pests.

## Conclusion

We found that urban bats prey mainly on agricultural pests. However, they also use insect species, such as Culicidae, which cause disease in humans such as malaria, filariasis, encephalitis, yellow fever, and dengue. The agricultural pests of the main crops are present in the diet of bats. In a conservative estimate, we found that bats can provide savings of US $ 390.6 million per harvest in maize crops. The results of this work show the importance of maintaining bat populations and their essential ecosystem services. With this information about bat pest suppression we hope to contribute for bat conservation in Brazil and elsewhere.
